# Burden assessment in caregivers of patients with schizophrenia

**DOI:** 10.1192/j.eurpsy.2025.1462

**Published:** 2025-08-26

**Authors:** W. Bouali, W. Mejai, R. Ben Soussia, A. Hadj Mohamed

**Affiliations:** 1 psychiatry, mahdia hospital, mahdia, Tunisia

## Abstract

**Introduction:**

The role of caregiver of patients with schizophrenia is associated with an increased risk of adverse mental and physical health outcomes.

**Objectives:**

assess the severity of the burden in caregivers of patients with schizophrenia as well as to determine the factors associated with a high burden with the overall goal of improving the quality of life of caregivers of patients with schizophrenia.

**Methods:**

This is a descriptive mono-centric cross-sectional study with an analytical aim carried out during the period March-April 2022 on 80 caregivers of schizophrenia patients followed up at the psychiatric consultations of the University Hospital of Mahdia. The evaluation of the burden experienced by the caregiver was carried out using the “SCQ” Schizophrenia Caregiver Questionnaire Version 1.0.

**Results:**

The average age of the caregivers was 57.1 years with 54% of them being male. 71% of the caregivers were married with 81% of them being educated and 19% being illiterate. 45% of the caregivers were still active with 58% of them having an average socio-economic level. Parents represented 59% of caregivers, siblings 29% and spouses 14%. They lived with the patients in 96% of cases and took care of another family member in 37.5% of cases. 89% of the caregivers were taking care of the patients for a period of more than 5 years and in 77.5% of the cases, these caregivers requested specialized help. 75% of patients were completely dependent on the caregiver.

37.5% of caregivers had a severe level of burden. Four factors were found to be predictive of severe burden: low education level of the caregiver, low socioeconomic level of the caregiver, violent and aggressive behaviors of the patient, and degree of dependence of the patient (p<0.05).

**Conclusions:**

Being a caregiver of a patient with schizophrenia is correlated with a great burden and deterioration of quality of life, hence the importance of assessing this burden, determining the initiating and aggravating factors as well as developing well-defined strategies for these caregivers in order to alleviate this burden and improve their quality of life.

**Disclosure of Interest:**

None Declared

